# Toward radiomics for assessment of response to systemic therapies in lung cancer

**DOI:** 10.18632/oncotarget.27847

**Published:** 2020-12-22

**Authors:** Shawn Sun, Florent L. Besson, Binsheng Zhao, Lawrence H. Schwartz, Laurent Dercle

**Affiliations:** ^1^Department of Radiology, New York Presbyterian Hospital, Columbia University Medical Center, New York, New York, USA; ^2^Department of Biophysics, Nuclear Medicine-Molécular Imaging, Hôpitaux Universitaires Paris-Saclay, AP-HP, Université Paris Saclay/CEA/CNRS/Inserm/BioMaps, Paris, France

**Keywords:** positron emission tomography, computed tomography, prognosis, lung cancer, immunotherapy

## Abstract

This editorial comment explains recent developments in radiomics regarding the use of quantitative imaging biomarkers to predict lung cancer sensitivity to a variety of cancer therapies. Tumor response assessment has been a crucial component guiding cancer treatment. Evaluation of treatment response was standardized and classically based on measuring changes in tumor lesion size. Recent breakthroughs in artificial intelligence pave the way for the use of radiomics in tumor response assessment. Such objective techniques would bring a remarkable transformation to conventional methods, which can be inherently subjective. Successful implementation of these technologies would allow for faster and more accurate predictions of treatment efficacy, which will be critical to the advancement of personalized medicine.

## EDITORIAL COMMENT

Lung cancer is the leading cause of cancer death worldwide [1]. Systemic treatment for lung cancer is a continuously evolving landscape with several options, such as cytotoxic chemotherapy, molecularly targeted therapy and immunotherapy [2]. Imaging is critical for the assessment of tumor response [3]. However, standard imaging metrics are limited when assessing response and progression with emerging targeted and immune therapies. A change in the size of a subset of target lesions, which remains the gold standard for assessing response under treatment [3, 4], does not or cannot fully capture the complexity of lung cancer behavior, including the primary tumor and metastases, driven by its high cellular heterogeneity [5]. In the era of precision medicine, the challenge entails choosing the right treatment for the right patient at the right time. The core concept is to personalize medical care and optimize cost-effectiveness. Recent updates of the Response Evaluation Criteria in Solid Tumors (RECIST) to iRECIST [6], irRECIST [7], and many others [8–10] have proposed novel patterns of tumor response and progression to immunotherapy. These updates begin to address part of the challenge, however, the paradigm of response assessment is shifting toward new imaging methods.

Radiomics -the use of imaging features from radiographic medical images transform images into quantitative data- has emerged a decade ago [11]. This high-throughput feature extraction procedure significantly increases the radiologist's analysis capability. However, despite the growing availability of dedicated software, numerous methodological challenges have to be faced with validating the procedure, which explains the slow implementation of such a revolutionary approach in clinical practice: the difficulty to collect massive structured imaging data from suitable target populations; the implementation and harmonization of operational multilevel imaging pipelines including imaging acquisition, tumor segmentation, feature extraction, and finally the feature selection process, adapted to a particular output task which requires AI-based validation procedures [12].

The recent study entitled "Identification of Non-Small Cell Lung Cancer Sensitive to Systemic Cancer Therapies Using Radiomics" offers a relevant clinical illustration of the current predictive capabilities of radiomics in NSCLC tumors to several systemic treatments [13]. Using standard-of-care CT-scan images (baseline and first-treatment assessment) of NSCLC patients collected from clinical trials, machine-learning algorithms were trained to predict NSCLC sensitivities to the following treatments: nivolumab, docetaxel, and gefitinib. To this end, this study combined Radiomic features derived from the largest measurable lung lesion of each patient. These radiomics signatures achieved areas under the receiver operating characteristic curve (AUC) of 0.77, 0.67 and 0.82, for nivolumab, docetaxel, and gefitinib, respectively. The radiomic features used in these signatures characterized 1) tumor burden, 2) tumor spatial heterogeneity, and 3) density change around tumor-parenchyma boundaries. Interestingly, the radiomic signatures in nivolumab (immunotherapy) and gefitinib (EGFR-targeted) arms were dominated by intra-tumor spatial heterogeneity and tumor-parenchyma density transition descriptors. In contrast, volume descriptors were more relevant in the docetaxel (chemotherapy) cohort. This proof-of-concept study paves the way for future use of radiomics and AI in tumor response assessment. Of note, the ultimate goal is to find a signature that could generalize to all treatment types.

**Figure 1:**
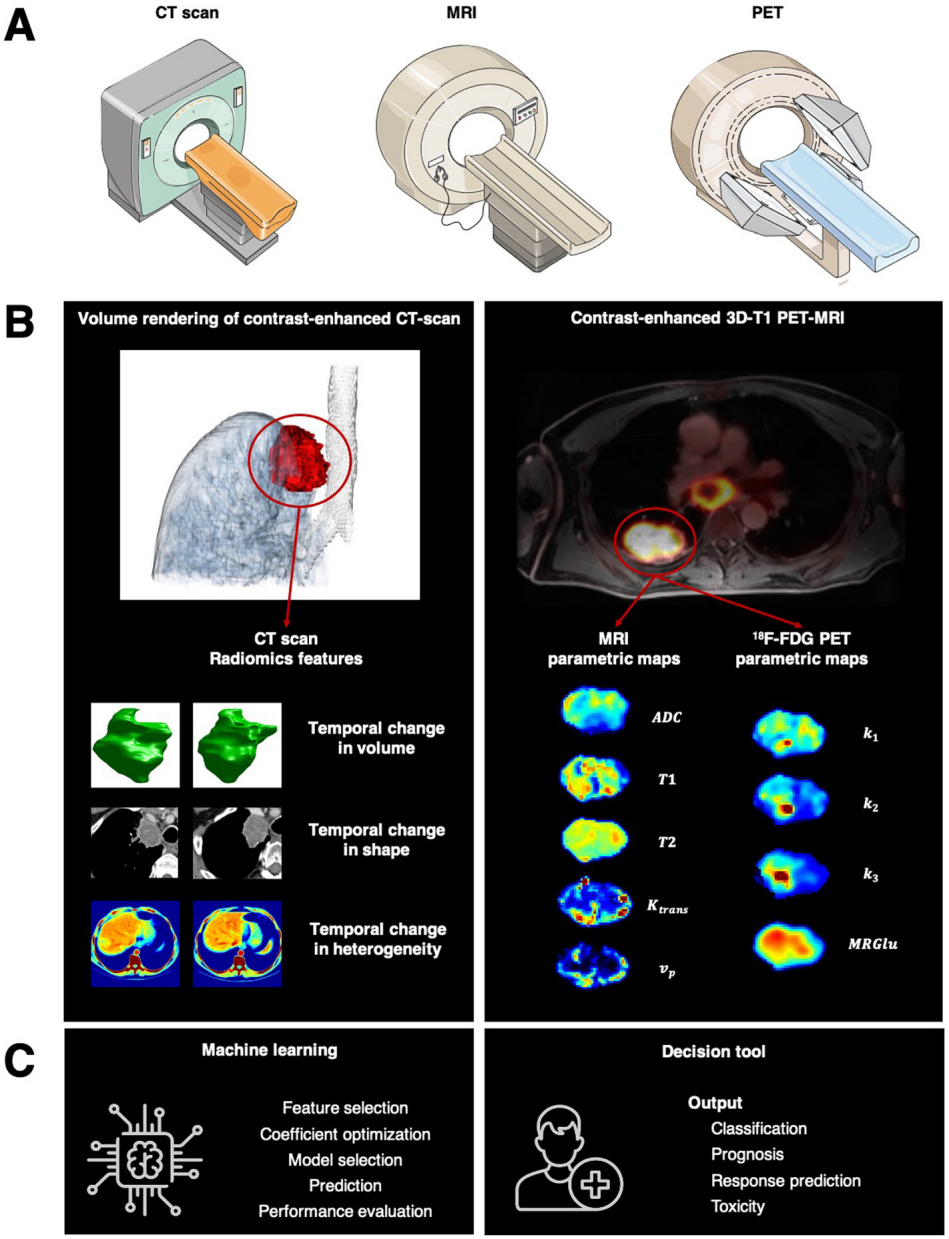
Toward radiomics for assessment of response to systemic therapies in lung cancer. (**A**) Multiple imaging modalities can characterize tumor imaging phenotypes such as CT scan, MRI, and PET. Multiparametric imaging offers unique opportunities to extend the image-based tumor analyses to more holistic characteristics. (**B**) left. In “Identification of Non-Small Cell Lung Cancer Sensitive to Systemic Cancer Therapies Using Radiomics,” the authors demonstrated that change over serial radiographic measurements in radiomics features deciphering tumor volume, invasion of tumor boundaries, or spatial tumor heterogeneity predicted tumor sensitivity to treatment, offering an approach that could enhance clinical decision-making to continue systemic therapies and forecast overall survival. (B) right. A fundamental breakthrough would be integrating multiparametric imaging data into the radiomic framework, such as imaging features extracted from functional MRI and 18F-FDG PET. (**C**) Machine-learning approaches can unravel among these imaging features, new imaging biomarkers predicting tumor sensitivity to treatment.

In perspective, a fundamental breakthrough would be to integrate multiparametric imaging data into the radiomic framework (Figure 1). Multiparametric imaging offers unique opportunities to extend the image-based tumor analyses to more holistic characteristics [14], including both non-parametric (qualitative) and parametric (quantitative) imaging data [15, 16]. A multiparametric radiomics signature could be a suitable combination of features extracted from non-fused or fused multimodal data [17, 18] or higher-order composite vector representation of the tumor temporal and spatial changes between multidimensional features [19, 20]. This is a new era to investigate the combination of imaging features extracted through various methods to guide clinical care. AI techniques would bring a remarkable transformation and redefine medical image interpretation from an inherently subjective human-based interpretation to an objective computer-based pattern-recognition algorithm. Successful implementation of these technologies would allow for faster and more accurate treatment efficacy predictions, which will probably significantly impact clinical outcomes and decision-making.
